# Modeling the Patient Journey from Injury to Community Reintegration for Persons with Acute Traumatic Spinal Cord Injury in a Canadian Centre

**DOI:** 10.1371/journal.pone.0072552

**Published:** 2013-08-30

**Authors:** Argelio Santos, James Gurling, Marcel F. Dvorak, Vanessa K. Noonan, Michael G. Fehlings, Anthony S. Burns, Rachel Lewis, Lesley Soril, Nader Fallah, John T. Street, Lise Bélanger, Andrea Townson, Liping Liang, Derek Atkins

**Affiliations:** 1 Rick Hansen Institute, Vancouver, Canada; 2 Fraser Health Authority, Surrey, Canada; 3 Division of Spine, Department of Orthopaedics, University of British Columbia, Vancouver, Canada; 4 Department of Surgery and Spinal Program, University of Toronto, Toronto, Canada; 5 Division of Physiatry, University of Toronto, Toronto, Canada; 6 Centre for Operations Excellence, Sauder School of Business, University of British Columbia, Vancouver, Canada; 7 Vancouver General Hospital, Vancouver, Canada; 8 Division of Physical Medicine and Rehabilitation, University of British Columbia, Vancouver, Canada; 9 Faculty of Business, Lingnan University, Tuen Mun, New Territories, Hong Kong; University of South Florida, United States of America

## Abstract

**Background:**

A patient’s journey through the health care system is influenced by clinical and system processes across the continuum of care.

**Methods:**

To inform optimized access to care and patient flow for individuals with traumatic spinal cord injury (tSCI), we developed a simulation model that can examine the full impact of therapeutic or systems interventions across the care continuum for patients with traumatic spinal cord injuries. The objective of this paper is to describe the detailed development of this simulation model for a major trauma and a rehabilitation centre in British Columbia (BC), Canada, as part of the Access to Care and Timing (ACT) project and is referred to as the BC ACT Model V1.0.

**Findings:**

To demonstrate the utility of the simulation model in clinical and administrative decision-making we present three typical scenarios that illustrate how an investigator can track the indirect impact(s) of medical and administrative interventions, both upstream and downstream along the continuum of care. For example, the model was used to estimate the theoretical impact of a practice that reduced the incidence of pressure ulcers by 70%. This led to a decrease in acute and rehabilitation length of stay of 4 and 2 days, respectively and a decrease in bed utilization of 9% and 3% in acute and rehabilitation.

**Conclusion:**

The scenario analysis using the BC ACT Model V1.0 demonstrates the flexibility and value of the simulation model as a decision-making tool by providing estimates of the effects of different interventions and allowing them to be objectively compared. Future work will involve developing a generalizable national Canadian ACT Model to examine differences in care delivery and identify the ideal attributes of SCI care delivery.

## Introduction

Spinal cord injury (SCI) can result in irreversible neurological impairment of the motor, sensory and/or autonomic nervous systems; and was estimated to affect 85,556 persons in Canada in 2010 [1]. Traumatic spinal cord injuries (tSCI) occur from external impacts such as motor vehicle collisions, sports accidents, falls, or violence and account for over half of the affected population, with an estimated prevalence of 43,974 or 1,298 per million in Canada in 2010 and approximately 1,785 new cases each year [1]. Nearly half of all traumatic injuries are due to damage of the cervical spinal cord (referred to as tetraplegia), with the remainder occurring in the thoracic, lumbar and sacral spinal cord (referred to as paraplegia) [2]. While approximately 80% of new injuries continue to occur among men, the average age at injury has been increasing, rising from 28.3 years in 1970 to 37.1 years in 2005 in the United States [3] and a bimodal distribution is being reported internationally, with the first peak between 15 and 29 years and a second peak after 50 to 65 years [2], [4].

Care for patients with tSCI demands a number of resources such as hospital trauma care, rehabilitation and then typically lifelong follow up. The estimated incidence of tSCI in Canada is relatively low compared to conditions such as stroke or cardiac disease. The economic impact of this condition, however, is high due to patient’s frequently young age and the profound disability [5]. Advances in tSCI care have resulted in increased survival from the trauma and patients experiencing a close to normal life expectancy [6], [7]. Care delivery standards in pre-hospital, acute and rehabilitation settings are emerging, but significant variation among centres remains.

We distinguish a patient’s journey through the health care system as influenced by clinical *and* system (administrative) processes and decisions. Although somewhat imprecise, clinical processes can be thought to *directly* impact patient outcomes whereas system processes involve resources, some degree of specialization, staffing and location-all of which may impact length of stay, time to surgery or rehabilitation intensity, thus *indirectly* affecting patient outcomes. Clinical and system decisions are tightly integrated for patients with tSCI as they receive care in different units and require multiple therapeutic interventions over time. This tight integration implies that, to properly evaluate the full impact of interventions in terms of both improving efficiency and the quality of care, a better understanding of patient flow across the continuum of care is required. To gain this understanding, there is a need to predict, track and evaluate the results of such interventions [8], [9].

This paper describes an experiment in modeling long-term patient flow to simulate interventions under different conditions, testing various ‘what-if’ hypotheses. This is particularly useful if a system is extremely complex or when real system testing is costly or impossible. Discrete event simulation (DES), the methodology employed for this study, has been used in other health care fields including: patient flows in intensive care units [10], emergency departments [11]–[13], medical out-patient clinics [14] and cost-effectiveness of hip and knee replacement [15]. However, we are not aware of any attempt to model the end-to-end continuum of care for a condition such as tSCI including pre-hospital, acute, rehabilitation and community phases. In 2010, we initiated the Access to Care and Timing (ACT) project, a multicentre study to model the continuum of SCI care in Canada [16]. The goals of ACT are to model the delivery of care throughout the SCI continuum; to simulate administrative, policy and therapeutic initiatives; and to predict and measure their influence on system costs and patient outcomes [16]. The objective of this paper is to describe the detailed development of a simulation model of the continuum of tSCI care for a major trauma and rehabilitation centre in Vancouver, British Columbia (BC), Canada, known as the BC ACT Model V1.0, which served as our pilot site for ACT. This paper presents the data requirements, decision-making processes for model development and, importantly, a few sample “what-if” scenarios and their possible effects on the entire system of care, comparing metrics of interest on a patient level (e.g. neurological recovery, incidence of secondary complications) and system level (e.g. length of stay, bed utilization)using data from BC.

## Methodology

### Study Design

**Figure 1:**
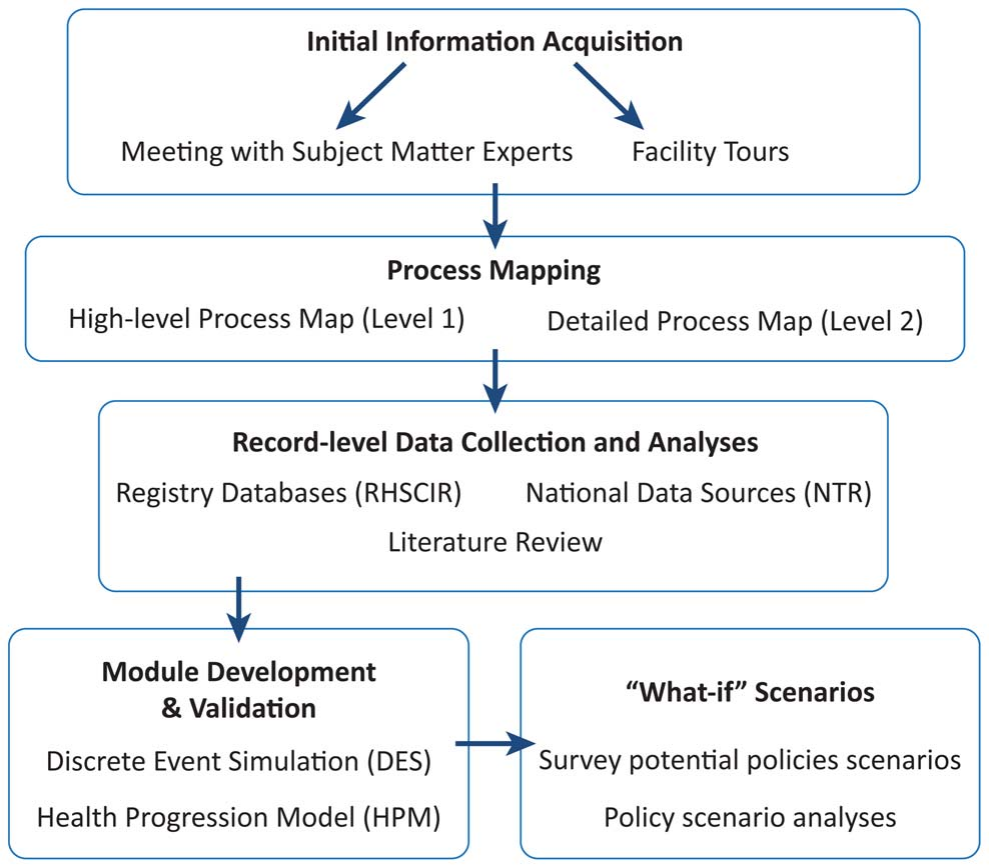
Flow chart of the study design. Starting with initial information acquisition, followed by process mapping (level 1 and 2), data collection & analyses, model development & validation and “what-if” scenario analyses.

Operations research techniques, specifically process mapping and discrete-event simulation (DES) modeling, were employed to develop a computer simulation of patient flow for tSCI through the continuum of care (pre-hospital, acute care and rehabilitation) to discharge into the community. We obtained ethical approval for this study from the University of British Columbia and the Vancouver Coastal Health Research Institute. The project was carried out by first meeting with subject matter experts and conducting site visits of the acute and rehabilitation facilities. A high-level process map was then generated to describe the care settings, resource availability, and services provided at the acute care and rehabilitation facilities; a second, detailed process map was subsequently developed to describe decision points and criteria for decisions which may affect the flow of patients with tSCI through the continuum of care. Prospectively collected, de-identified record-level data obtained from the local Vancouver Rick Hansen Spinal Cord Injury Registry (RHSCIR) [17] was analyzed for this study and a DES model was developed using the two process maps and the data analyses as inputs (see Figure 1 for a flow chart of the study design). Once validation of the model was completed, experimentation with “what-if” scenarios was done to evaluate the potential impact of different interventions across the entire continuum of care.

### Study Sample

The primary record-level data source for this study is the local Vancouver Rick Hansen Spinal Cord Injury Registry (RHSCIR), which contains 532 patients who were admitted to either the Vancouver General Hospital (VGH) (n = 174), GF Strong Rehabilitation Centre (n = 31) or both (n = 327) between April 2004 and July 2009. The RHSCIR is a longitudinal observational registry that collects demographic and clinical data on patients with tSCI in the pre-hospital, acute, rehabilitation and community settings from 31 sites across Canada. Following discharge from a RHSCIR site, a community follow-up questionnaire is also administered to participants at 1, 2, 5, and then every 5 years post-injury [17] if they have provided consent.

For specific data elements, the number of missing values in the Vancouver RHSCIR ranged from 0 to 255 (48%).

### Study Setting and Patient Flow for Acute Traumatic Spinal Cord Injury

#### Pre-Hospital phase

In the province of BC (population 4.5 million), the BC Ambulance Service responds to and transports trauma patients (including tSCI) injured within a 40 minute travel distance (by road) of a trauma-receiving hospital, directly to that hospital, bypassing any non-trauma hospitals. Patients injured more than 40 minutes away from a trauma hospital are often taken to the closest hospital for stabilization and afterwards transferred to a trauma hospital. The Air Ambulance protocol known as “Autolaunch” also expedites transfer to a trauma centre. BC has six accredited trauma centres, but only one has a designated Acute Spine Program (see next section) to which paramedics can transfer patients directly if paralysis is suspected at the scene of injury.

#### Acute care phase

The Acute Spine Program is located at VGH, which is the only quaternary care centre for the province of BC [2]. The Acute Spine Program at VGH and GF Strong (see next section) are also participating sites in the RHSCIR [17]. According to the Vancouver RHSCIR database, there were 501 tSCI patients admitted to VGH between the 5-year period of April 2004 and July 2009.

As a Level 1 trauma centre, VGH admission policies ensure that patients with serious medical injuries are guaranteed admission. Patients with suspected tSCI are admitted through the emergency department and consulted by surgeons affiliated with the VGH Acute Spine Program. Following assessment and imaging, patients go directly to surgery, the intensive care unit or the spine ward. During their stay, patients might receive repeat surgeries or intensive care unit admissions depending on the injury or adverse events.

The VGH Acute Spine Program has six spine surgeons (neurosurgeons and orthopaedic surgeons), plus nursing and allied health staff with specific expertise in SCI management.

#### Rehabilitation phase and community living

The largest rehabilitation centre in BC is GF Strong, located approximately two kilometres from VGH. GF Strong has a SCI Program, accepting both in- and out-patients from VGH, amongst other acute care facilities. In 2010, GF Strong admitted approximately 143 in-patients (including readmissions), with 67% (96 cases) being tSCI. Patients are consulted by a physiatrist prior to admission to confirm appropriateness for rehabilitation. Patients requiring imaging or invasive treatment are re-admitted to VGH. The SCI Program at GF Strong has five physiatrists and an interdisciplinary team with expertise in SCI rehabilitation and care. Patients have access to specialized services including sexual health, seating clinics, vocational rehabilitation, psychology, urology, and respiratory therapy.

Following discharge from GF Strong, individuals with tSCI may return home, often after extensive home and vehicle modifications, or may go to a long-term care facility or a group home. Many individuals with tSCI require out-patient rehabilitation to assist with attaining rehabilitation goals or to address health issues that may arise subsequent to their initial in-patient care. The life expectancy of persons with tSCI is somewhat shorter than for the general population [18], depending on many factors including age at injury and the severity of their injury [19]. At age 40 the life expectancy of an uninjured person is estimated at 39.9 years, but for a person living with a tSCI the life expectancy ranges from 33.9 years to 8.0 years depending on the severity of injury [18].

### Statistical Analyses

The type of regression methodologies used depended on the distribution of response variables and their statistical properties: for continuous data exhibiting a normal distribution we used multiple linear regression; whereas for categorical outcomes multinomial logistic regression or ordinal logistic regression were used.

Two different R-square measures were used to evaluate goodness of fit, namely pseudo R^2^ and Max-rescaled R^2^. They are likelihood-based and were used to measure goodness of fit and should not be interpreted as the proportion of variation in the dependent variable. Max-rescaled R^2^ is defined as pseudo R^2^ divided by its maximum and was used when there was at least one dichotomous independent variable. Also, the Wald Chi-Square test was used to test the statistical significance of parameters in the logistic regression models.

Treatment of missing data depended on the situation. First, the regression analysis was done including dummy variables for the missing records, and if the missing records were not statistically significant in predicting the response variable, then those records were excluded from the analysis. For some variables with missing records the statistical analysis was not performed and results from the medical literature were used in the ACT model. Imputation using statistical analysis was also conducted for the analysis of length of stay in the acute and rehabilitation facilities; the imputation was done for the independent variables that had previously been analyzed excluding their missing records [e.g. neurological classification determined by the American Spinal Injury Association (ASIA) Impairment Scale [AIS]].

Statistical analyses were performed using SAS® software (version 9.3; SAS institute, Cary, NC).

## Results

### Development of the Care Continuum Patient Flow Model for Traumatic SCI

The ACT model design reflects four functional specifications. A: *Evaluative.* It must demonstrate how interventions, such as additional resources, or clinical decisions, such as increasing the number of injured patients going directly to a specialized centre, impact the continuum of care. Impact refers not only to direct impacts, but also to second and third order impacts (i.e. an impact chain where cause 1 yields impact 1; then impact 1 becomes cause 2 which yields impact 2 (second order impact) and so on). B: *Quantitative*. Such impacts must be quantifiable to sustain evidence-based decision making. C: *Transparent, Portable and Flexible*. Clear documentation of data sources (e.g.: RHSCIR data or published literature); wherever possible, we used international standards for data elements. Flexibility is characterized by responsiveness to changing information. As data accumulates, new studies are published or organizational changes occur, the model must be adaptable. D: *Accessible and Maintainable*. The model must be understandable to clinicians and staff typically found in clinical centres of excellence.

Four main data sources populate the model:

1. The RHSCIR data which currently contains over 3,000 Canadian patient records (532 records from BC at the time of this study). RHSCIR data included in the simulation are from the patients’ journeys through pre-hospital, acute, rehabilitation and community follow-up outcomes [17];
2. A comprehensive literature review comprising summaries or data extracts from over 300 manuscripts;
3. Subject Matter Experts’ knowledge about processes and procedures. Subject matter experts are characterized as individuals with content expertise in a specific phase of the continuum of tSCI care, because of clinical role or other profession. For example, a paramedic advising on the pre-hospital phase, an intensive care unit nurse advising on processes in the intensive care unit and a social worker from the rehabilitation centre able to describe the decision-making process for discharge into the community, were all considered as subject matter experts for this study.
4. Other data sources such as the Canadian Institute for Health Information’s National Rehabilitation Reporting System which includes data from rehabilitation centres across Canada.

### The Architecture of the ACT Model: Simulating Patient Flow

**Figure 2:**
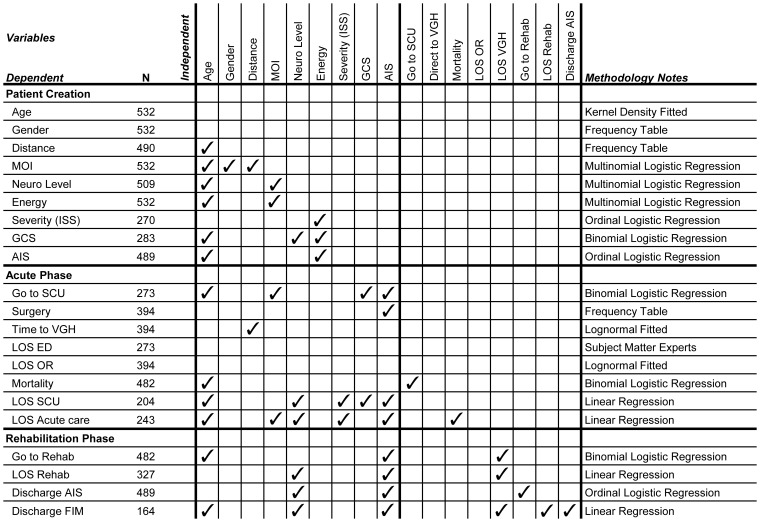
Summary of the variables estimated and the methodology involved in creating the simulation model. All variables are significant (p-value<0.05). ✓Indicates the independent variable(s) that can estimate the dependent variable (e.g. the dependent variable AIS can be estimated by the independent variables age and energy); MOI: Mechanism of Injury; Neuro Level: Neurological Level of Injury; ISS: Injury Severity Score; GCS: Glasgow Coma Scale; AIS: ASIA Impairment Scale; SCU: Special Care Unit; VGH: Vancouver General Hospital; LOS: Length of Stay; ED: Emergency Department; OR: Operating Room; Rehab: Rehabilitation; FIM: Functional Independence Measure.

The DES component of the ACT model was developed using ExtendSim© (Imagine That Inc. San Jose, CA) software and tracks individual simulated ‘patient’ entities from first response to community living. As simulated patients flow from the location of injury to acute care and through to rehabilitation, they consume resources and accumulate properties to each patient record. These properties may include therapeutic episodes (surgery), length of stay, adverse events such as secondary complications (pneumonia, pressure ulcer, etc.) and changes in neurological impairment, in essence creating a simplified ‘patient chart’ that, similar to a real chart, grows with events. We describe each unit in Figure 2. These patient properties are the outputs of the simulation, used to compare the impact of changes in the system (e.g. comparison of ‘time to surgery’ before and after adding resources in the pre-hospital phase) and those properties most impacted are then used to create a flow diagram for ease of presenting the overall results to clinicians and decision makers.

To incorporate the four data sources described, we first met with various clinical experts in tSCI care to understand the patient flow, staffing and resources for each phase of care represented in the model. Based on the information gathered from these meetings, we developed detailed process maps that depicted patient flow and clinical decision making throughout the continuum of care. We then conducted univariate and bivariate analyses to obtain a general ‘picture’ of the data before starting the regression analysis.

Figure 2 shows the significant variables (p<0.05) at each step, but does not show the actual coefficient value because of the wide range of regression methodologies employed.

From consultation with clinical experts, neurological classification at the time of discharge (discharge AIS) was identified as an important outcome. We used an ordinal logistic regression model (Table 1) to describe changes in the individual status of initial AIS to discharge AIS. In the multivariable analysis, baseline performance of AIS was significantly associated with the performance of discharge AIS (i.e. the less severe the initial AIS, the greater the probability of neurological improvement). Importantly, neurological level of injury had significant effects on changes in AIS as well (i.e. a greater proportion of individuals with thoracic and lumbar injuries were associated with increased injury severity, and are less likely to exhibit neurological improvement compared to individuals with cervical injuries). The remaining models used in the simulation are available in the supplementary data (see Tables S1 to S15 in File S1).

**Table 1:** Ordinal logistic regression results for discharge AIS (number of observations = 489).

Parameter	DF	Estimate	Standard Error	Wald Chi-Square	P-value
Intercept (E)	1	−3.036	0.332	83.824	<.0001
Intercept (D)	1	2.854	0.363	61.639	<.0001
Intercept (C)	1	4.107	0.402	104.465	<.0001
Intercept (B)	1	5.769	0.438	173.099	<.0001
Neurological Level (Low)	1	−0.454	0.228	3.943	0.0471
Admission AIS (A)	1	−7.311	0.491	221.436	<.0001
Admission AIS (B)	1	−4.996	0.494	102.421	<.0001
Admission AIS (C)	1	−2.768	0.442	39.227	<.0001
Rehabilitation (Yes)	1	1.157	0.254	20.735	<.0001

R-Square = 0.6637,Max-rescaled R-Square = 0.7156.

Dependent variable: AIS at Discharge (A, B, C, D, E).

Independent variables: AIS at Admission (A, B, C, D), Neurological Level (High, Medium, Low), Rehabilitation (Yes, No) AIS: American Spinal Injury Association (ASIA) Impairment Scale DF: degrees of freedom.

The Wald Chi-Square test is used to test the statistical significance of parameters in the logistic regression models.

### Patient Creation and First Response

Using demographic data from RHSCIR, incidence rates were estimated, adjusted for day of the week and month to generate the injury time/date. Descriptive data from the Vancouver RHSCIR was used to create simulated patients with the appropriate age, gender and distance from scene of injury to hospital. An integrated chain of various regressions, summarized in the top section of Figure 2, generated correlated probabilities for assigning patient properties. For example, age, gender and distance to VGH (as a proxy for rural versus urban areas) were significant explanatory variables for the probability of mechanism of injury, which includes transport, sports, falls, and other causes of traumatic injury (Figure 2). This patient creation stage generated simulated patients differentiated into more than 640 distinct ‘profiles’ (age, gender, mechanism of injury, AIS, etc.).

The pre-hospital protocols applied to the simulated patients determined whether the patient went directly to VGH, the time to admission to VGH (conditioned on patient properties) or whether the patient traveled first to another hospital. The mode of transport (road ambulance or fixed or variable wing aircraft) was implied by the location of the injury, the severity and the time of admission to VGH. At the end of this stage, the ‘admitted patient entity’ has a personal profile and injury characteristics along with information about the time and route taken to VGH.

### Acute Care at VGH Acute Spine Program

This complex module included the emergency department, operating room, special care unit – which is essentially an intensive care unit at VGH-and finally an acute in-patient spine ward. Three types of events were added to the patient record during acute care: surgery, secondary complications, and length of stay. For those patients assigned to have surgery, a time to surgery, time in the operating room, probability of mortality, and time in the special care unit after surgery were generated. These data came primarily from RHSCIR (mainly estimated via logistic regressions) with some subject matter expert advice on critical information not captured in RHSCIR (Figure 2, second section).

Assigning secondary complications, or adverse events, was important but challenging to quantify. RHSCIR does not currently collect prospective incidence data on secondary complications; therefore the primary information source was from the literature. Typical odds ratio models can certainly consider dependence among explanatory variables such as age and neurological level using cross variables (e.g.: gender*neurological level) but this approach quickly exhausts the available data. Instead, incidence estimates of complications such as pressure ulcers, neuropathic pain, pneumonia, urinary tract infections and delirium (identified as the five most frequent secondary complications for patients with tSCI admitted to the VGH Acute Spine Program [20]) were made through a novel least squares minimization constrained by results from the literature. Thus, for each of the possible 640 patient profiles, a probability of getting one of these five complications was needed, totalling 3,200 probabilities. An example of the type of constraint drawn from the literature, and included in the minimization, is that males have twice the chance of females of getting a pressure ulcer [21]. Evidence in the literature related to the incidence of secondary complications became a constraint in the model. As new published evidence becomes available, it can be encoded as a new constraint and the minimization re-run, automatically updating simulation probabilities. However, until more site-specific data becomes available these complication probabilities must remain generic.

Length of stay at VGH was estimated from a combination of subject matter expert sources and RHSCIR data. The last step in the acute care phase is to track patients who are clinically ready (i.e. no further acute care needs) for discharge to the appropriate destination (e.g. rehabilitation, home) but who, for various reasons, were unable to be discharged. This undesirable outcome is caused by lack of appropriate capacity downstream and is termed ‘alternative level of care’.

### In-Patient Rehabilitation at GF Strong

Simulated patients transition to rehabilitation with all their properties assigned during pre-hospital and acute care phases. Complications in rehabilitation were assigned based on frequencies gleaned from data on service interruptions obtained from the Canadian Institute for Health Information National Rehabilitation Reporting System, associated with their particular profile [18]. However, for some complications (e.g. pressure ulcers and urinary tract infections), previous exposure during acute care increased the patients’ predisposition to a recurrence. The evidence for such a predisposition, obtained from the Canadian Institute for Health Information National Rehabilitation Reporting System data, is not strong, despite anecdotal evidence from the subject matter experts, and further research is required.

During rehabilitation, re-admission to VGH because of a complication is possible. Although professional practice differs, a patient’s bed at GF Strong is ‘held’ for up to three days; after that, the patient is added to the wait list for rehabilitation.

Finally, some patients experience another alternative level of care queue in rehabilitation waiting for an appropriate discharge destination, such as their home modified to be accessible or other specialised housing.

### Long-term Outcome Measures

#### In-hospital cost

As each patient entity leaves the DES, their acute cost is based on total days in care, whether acute or rehab. Values for the per-admission and per-day costs in Canada were derived from a recent study which calculated their costs based on the available literature and health economic methodology [22].

#### Life expectancy

Life expectancy at discharge is based on age, neurological level and AIS. Life years lost were calculated through age-comparison with uninjured people.

#### Long-term costs

Long-term costs, one-time cost (e.g. home modifications) plus yearly costs (e.g. physician visits), were based on life expectancy, neurological level and AIS; and were calculated for each individual [22] following community reintegration.

#### Quality-adjusted life years lived

The RHSCIR dataset provides participant responses from the Short Form-36 (SF-36) and a complications survey completed at regular follow-up time points. The SF-36 includes 36 questions that measure functional health and well-being from the patient’s point of view; it provides scores for eight health domains (physical functioning, role-physical, bodily pain, general health, vitality, social functioning, role-emotional and mental health) [23]. The SF-6D algorithm was used to calculate utility values from the SF-36 responses [24]. These utility values were regressed on predictors including age at injury, gender, neurological level, AIS at discharge, the physical domain of the Functional Independence Measure [25] (which is an instrument used to assess physical and cognitive function in patients) at discharge, and the presence of complications at follow-up. Only neurological level (cervical vs. non-cervical) and three complications (urinary tract infections, neuropathic pain, depression) were significant. The probabilities of complications being present were also calculated: for those three aforementioned complications there were no significant covariates within the RHSCIR dataset, thus equally likely among all people.

The model calculated quality-adjusted life years (QALYs) in a number of different ways, to account for different possible future sets of complications: (i) assuming no complications in any future year, (ii) assuming all three complications in all future years, (iii) assuming ‘average’ complications in all years (i.e. the probability of all episodes of urinary tract infections, neuropathic pain and depression were represented as an average by year), (iv) ‘average’ neuropathic pain and depression and no urinary tract infections and finally (v) ‘average’ neuropathic pain and depression and always incurring urinary tract infections if the person had had a urinary tract infection before discharge. Comparing these values for QALYs gives a way of assessing the potential future value of avoiding complications during acute or rehabilitation.

#### Costs due to complications

From the probabilities of urinary tract infections and pressure ulcers we assigned ‘average’ complications each year, and their associated costs based on the best available literature [22]. Since these costs were already included within the overall cost of treating SCI, they do not represent additional costs, but rather the portion of the total cost that could be avoided by specifically preventing pressure ulcers and urinary tract infections in the community.

### Technical Input-output Functionality

A spreadsheet interface loads all inputs (statistical calculations and parameters) and receives results. Evaluating interventions or policy scenarios employs 50 replications, each of which includes a five year warm-up period to allow the simulation to stabilize based on the ExtendSim output, and then five years of collecting output metrics, including expected values and 95% confidence intervals.

### Model Validation

Validation included standard statistical validation for individual estimations (over 50 regressions/estimations), using an independent cross-validation dataset wherever possible and an overall validation of each module of the DES comparing its results against RHSCIR data. Further validation included a review of real and simulation-generated data to a subject matter expert to identify important mismatches that could have been missed by the statistical validation. With a sufficient number of new patients added to the dataset feeding the model, a limited number of tests have been compared against new data, not used in the ACT model development.

### Sample “What-if” Scenarios

We demonstrate the utility of this model with three proposed scenarios.

#### Scenario 1

**Figure 3:**
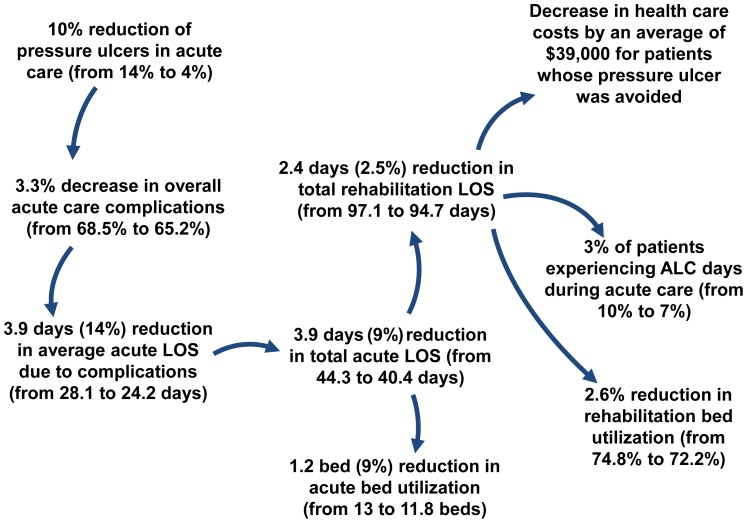
Scenario One: Indirect impact of pressure ulcer reduction during acute care. Reducing pressure ulcers during acute care has direct impacts on overall complications and length of stay in acute care, but also indirect impacts on rehabilitation complications, length of stay and bed utilization.

Three issues around pressure ulcers have wide agreement: they are common, they are ‘expensive’ in terms of costs, medical resources and patient risk, and they are partially preventable. However, the degree of agreement on each point varies widely. By utilizing the BC ACT Model V1.0, and following the advice from subject matter experts, we investigated the consequences of simulating a 70% reduction in the incidence of pressure ulcers in acute care. The ACT model does not presently track pressure ulcers by grade, so this is a 70% reduction for all pressure ulcer grades. Figure 3 shows that in addition to the direct effect, a cascade of other benefits is triggered. The probability of suffering from additional pressure ulcers during rehabilitation is reduced purely by removing the predisposition. There is a reduction in the average length of stay of 4 days (from 44 to 40 days) in acute care and 2 days (from 97 to 95 days) in rehabilitation care, allowing for enhanced bed availability in both acute by 9% (from 13 to 11.8 beds used) and rehabilitation by 3% (from 74.8% to 72.2% used beds out of 24 beds available). Patient outcomes in terms of health care costs are a $39,000 reduction per patient for persons who under the baseline scenario had a pressure ulcer and under the scenario 1 did not get a pressure ulcer. Overall, the business case for investment in any prevention program that could achieve a 70% success now looks significantly different by taking this full picture into account.

#### Scenario 2

**Figure 4:**
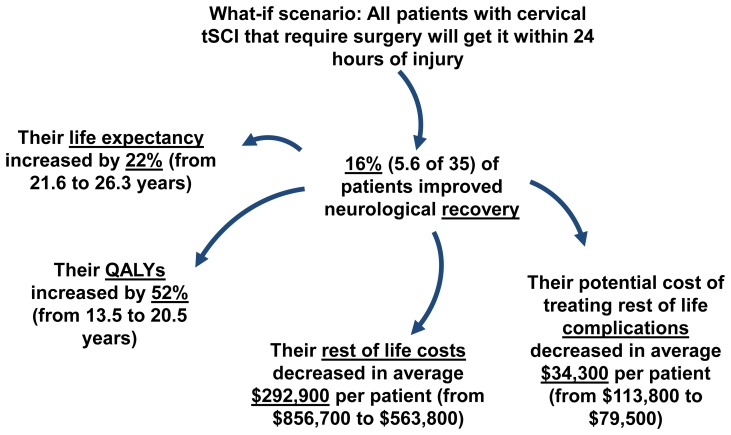
Scenario Two: Indirect impacts of early surgery. Providing early surgery to patients with tetraplegia has a direct impact on their neurological recovery and also indirect impact on their life expectancy, quality of life and savings in their rest of life costs.

Using the BC ACT Model V1.0 allows us to demonstrate additional indirect effects of early surgery (Figure 4). Running the model with the baseline scenario we obtain on average 100 patients per year with a tSCI; 35 of which would be affected by a policy change mandating surgery within 24 hours for cervical injuries. Results from the model demonstrated that, on average, 16% (5.6 of 35) of the patients had improved neurological recovery, almost a five year increase (from 21.6 to 26.3 years) in life expectancy and a seven year (from 13.5 to 20.5 years) increase in QALYs (Figure 3). The average savings in life costs was approximately a quarter of a million dollars and the potential lifetime costs of treating complications in the community also decreased by $34,400 (from $113,800 to $79,500) per patient (Figure 3).

#### Scenario 3

What if additional staffed beds were made available within the rehabilitation unit?

**Figure 5:**
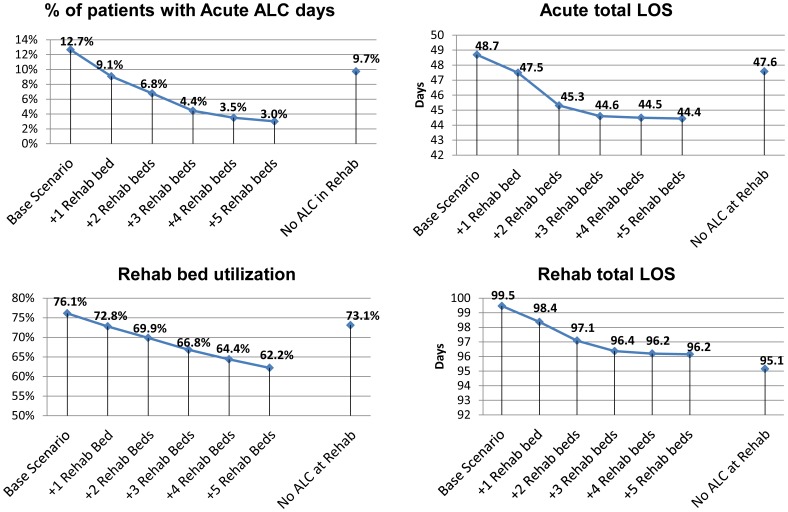
Scenario Three: The indirect impact of additional rehabilitation beds. Adding rehabilitation beds has an impact on the admission to rehabilitation waiting time (alternative level of care days in acute), acute length of stay, bed utilization at the rehabilitation centre and rehabilitation length of stay. ALC: Alternative Level of Care Days; LOS: Length of Stay; Rehab: Rehabilitation.

Figure 5 shows that adding two rehabilitation beds reduces the number of patients in acute care from 12.7% to 6.8% who are designated as appropriate for an alternative level of care (e.g. waiting for an available rehabilitation bed), which in turn decreases the total length of stay in acute care by 3 days (from 48.7 to 45.3). The length of stay at the rehabilitation centre is also reduced by two days (from 99.5 to 97.1 days), and even this small decrease impacts the utilization of rehabilitation beds, reducing it by 6% (76.1% to 69.9% occupancy).

## Discussion

The BC ACT Model V1.0 was developed using DES methodology as an experiment to evaluate the impact of clinical and administrative interventions along the entire tSCI continuum of care. Development of this model builds on existing studies that have used similar methodologies in health care research, but often only to examine a subset of outcomes in either a particular ward or phase of the continuum, such as resource utilization and planning in the emergency department [11]–[13], in the intensive care unit [26] or in the pre-hospital phase of care [27]. Other studies have also used a patient-level DES approach to model in-patient flow or pathways based on differing treatment modalities [28] or to describe progression through a given condition or disease state [29]. While these studies have similarly modeled some of the decision-making processes undertaken within various stages of care (e.g. emergency triage decisions, stratifying treatment groups), they do not directly simulate the facilities or hospitals that make up the end-to-end continuum, nor the complexity of the competing interactions and practices within and across those facilities.

Using the BC ACT Model V1.0, it is possible to model the entire continuum of care of tSCI and evaluate both system- and patient-related outcomes. Transitions between phases have been cited as a major challenge in providing optimal care for patients with tSCI and where clinicians are often constrained to working in silos [16]. The value in using a systems approach is that it enables the user to examine the potential impact(s) of interventions not just within the phase of care where it was applied (e.g. increased number of beds in an acute care ward) but to also consider the immediate and long-term implications upstream and downstream of the intervention. The BC ACT Model V1.0 can therefore serve as a tool to assist clinicians and decision-makers alike and provide the best available evidence for applications across the continuum.

The three scenarios demonstrate the flexibility and value of the BC ACT Model V1.0 as a decision-making tool. In Scenario 1 we show the indirect benefits of a clinical intervention on secondary complications, whereas in scenario 2, we show how another clinical intervention (early surgery) can also have additional, long-term benefits for the patients given a recent study [30] that has provided evidence that patients with a cervical SCI demonstrate significantly better neurological outcomes (by 2 AIS grades or more) if they receive surgery within 24 hours. In Scenario 3 we address questions of the up/downstream impacts of additional resources within the system. These experiments allow the model user to uncover a set of consequences that may be overlooked and not often collectively reconciled.

Apart from these applications, the work has demonstrated the value of partnerships where the participants bring different perspectives and expertise, in this case an academic business school and the accompanying application of business methodologies working together with two health care facilities and a research institute. Subject matter experts have commented that even the exercise of thinking through the patient flow was of value to them in seeing the patient journey as an integrated process. Existing knowledge gaps were also identified, highlighting important research that needs to be done to further refine care delivery. For example, the predisposition towards the recurrence of secondary complications in rehabilitation or the community following the initial occurrence of complications during acute care (e.g. pressure ulcer development) has been anecdotally identified by subject matter experts; however, there is limited published evidence for this.

Despite the apparent value of the BC ACT Model V1.0, there are still limitations that need to be considered. Full model validation using DES methods is an ongoing process and can be very time consuming and data intensive. As such, we anticipate that model validation will be an iterative and continuous process throughout the model development and use, especially if more sites wish to engage in the project and new data sources become available. In addition, the low incidence of tSCI and the extremely large variability between cases makes it difficult to obtain adequate data. This has highlighted the need to enhance the RHSCIR data used for the BC ACT Model V1.0 by including data from other Canadian provinces and create a Canadian ACT Model. In the meantime, there remains a need for advanced statistical analysis to deal with small sample sizes and missing records, and ongoing model validation, to ensure that the model reflects the best available data.

In summary, as part of the ACT project we have used DES to model the continuum of care for tSCI. The next steps in the study are to generalize and disseminate the model to other provinces. Specifically, we wish to engage a number of other sites with different facility structures (e.g. integrated acute care and rehabilitation facility), tSCI patient demographics, and clinical and administrative policies. We will undergo the same processes described in this paper for each of the subsequently added sites to ultimately create a generalizable Canadian ACT Model that will incorporate data from and facets of each of the models of care delivery. The variety in site types, and accompanying patient flows, will truly highlight the similarities and differences in the processes of care in Canada and create a stronger tool for clinicians and decision-makers across the country. Emerging evidence must continue to be reflected in the model. The hope is that results from this work will help inform the identification of best practices across the SCI continuum of care.

## Supporting Information

File S1 **This file contains Tables S1 through S15.** Table S1, Age Distribution. Table S2, Gender Distribution. Table S3, Regression Analysis Results for Mechanism of Injury. Table S4, Regression Analysis Results for Neurological Level. Table S5, Regression Analysis Results for Energy. Table S6, Regression Analysis Results for Injury Severity Score (ISS). Table S7, Regression Analysis Results for Glasgow Coma Scale (GCS). Table S8, Regression Analysis Results for ASIA Impairment Scale (AIS). Table S9, Regression Analysis Results for Go To SCU. Table S10, Regression Analysis Results for Mortality. Table S11, Regression Analysis Results for Length of Stay (LOS) in special care unit (SCU). Table S12, Regression Analysis Results for LOS in Acute Care. Table S13, Regression Analysis Results for Go to Rehab. Table S14, Regression Analysis Results for LOS in Rehabilitation Care. Table S15. Regression Analysis Results for Discharge FIM.
(DOC)
