# Supplementary material for: Modeling the Patient Journey from Injury to Community Reintegration for Persons with Acute Traumatic Spinal Cord Injury in a Canadian Centre
Source: PLoS One. 2013 Aug 30;8(8):e72552. doi: 10.1371/journal.pone.0072552 (PMC3758357; doi:10.1371/journal.pone.0072552)
Supplement: File S1 — This file contains Tables S1 through S15. Table S1, Age Distribution. Table S2, Gender Distribution. Table S3, Regression Analysis Results for Mechanism of Injury. Table S4, Regression Analysis Results for Neurological Level. Table S5, Regression Analysis Results for Energy. Table S6, Regression Analysis Results for Injury Severity Score (ISS). Table S7, Regression Analysis Results for Glasgow Coma Scale (GCS). Table S8, Regression Analysis Results for ASIA Impairment Scale (AIS). Table S9, Regression Analysis Results for Go To SCU. Table S10, Regression Analysis Results for Mortality. Table S11, Regression Analysis Results for Length of Stay (LOS) in special care unit (SCU). Table S12, Regression Analysis Results for LOS in Acute Care. Table S13, Regression Analysis Results for Go to Rehab. Table S14, Regression Analysis Results for LOS in Rehabilitation Care. Table S15. Regression Analysis Results for Discharge FIM. (DOC) [file pone.0072552.s001.doc]

**SupplemENTARY material**

In this section we provide the distributions of Age and Gender and the specifications of each regression model used.

**Table S1) Age Distribution**

Methodology:Kernel Density Fitted Distribution

Summary:

| N | Mean | Std Dev | Min | Max |
| --- | --- | --- | --- | --- |
| 532 | 43.61 | 19.60 | 15 | 91 |


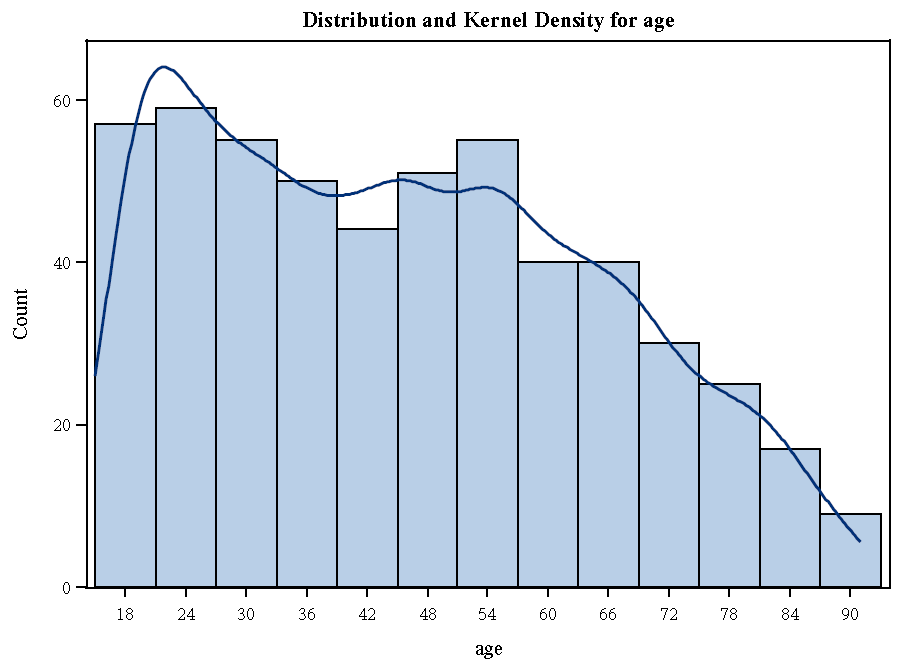


**Table S2) Gender Distribution**

Methodology:Frequency Table

Summary:

|  | Counts | Percentage |
| --- | --- | --- |
| Male | 427 | 80.3% |
| Female | 105 | 19.7% |

**Table S3) Regression Analysis Results for Mechanism of Injury**

Methodology:Multinomial Logistic Regression

Number of observations used:532

Dependent variable: MOI (Fall, Sports, Transport and Other)

Independent variables:

- Age
- Gender (Female, Male)
- Distance to VGH (Close, Medium, Far, Out Of Province and Unknown)

Results:

| Parameter | MOI | DF | Estimate | Standard Error | Wald Chi-Square | Pr>ChiSq |
| --- | --- | --- | --- | --- | --- | --- |
| Intercept | Fall | 1 | -1.5188 | 0.3856 | 15.5137 | <.0001 |
| Intercept | Other | 1 | 0.3837 | 0.5072 | 0.5724 | 0.4493 |
| Intercept | Sports | 1 | -0.2855 | 0.4657 | 0.3759 | 0.5398 |
| Age | Fall | 1 | 0.0516 | 0.00662 | 60.7185 | <.0001 |
| Age | Other | 1 | -0.0101 | 0.0105 | 0.917 | 0.3383 |
| Age | Sports | 1 | -0.0132 | 0.00803 | 2.6992 | 0.1004 |
| Gender_Female | Fall | 1 | 0.2058 | 0.274 | 0.5643 | 0.4525 |
| Gender_Female | Other | 1 | -2.0805 | 0.7555 | 7.5837 | 0.0059 |
| Gender_Female | Sports | 1 | -0.9519 | 0.3703 | 6.6079 | 0.0102 |
| DistanceToVGH_Medium | Fall | 1 | -0.8877 | 0.2853 | 9.6793 | 0.0019 |
| DistanceToVGH_Medium | Other | 1 | -1.4253 | 0.4119 | 11.9753 | 0.0005 |
| DistanceToVGH_Medium | Sports | 1 | 0.6498 | 0.3886 | 2.7959 | 0.0945 |
| DistanceToVGH_Far | Fall | 1 | -1.0687 | 0.3183 | 11.2704 | 0.0008 |
| DistanceToVGH_Far | Other | 1 | -1.6743 | 0.4751 | 12.4191 | 0.0004 |
| DistanceToVGH_Far | Sports | 1 | 0.1275 | 0.4234 | 0.0907 | 0.7633 |
| DistanceToVGH_Out | Fall | 1 | -2.086 | 0.526 | 15.7301 | <.0001 |
| DistanceToVGH_Out | Other | 1 | -1.9329 | 0.8006 | 5.8296 | 0.0158 |
| DistanceToVGH_Out | Sports | 1 | 0.4878 | 0.5175 | 0.8885 | 0.3459 |

| R-Square | 0.3097 | Max-rescaled R-Square | 0.337 |
| --- | --- | --- | --- |

**Table S4) Regression Analysis Results for Neurological Level**

Methodology:

Multinomial Logistic Regression

Number of observations used:

509

Dependent variable:

- Neurological Level (High, Medium and Low)

Independent variables:

- Age
- MOI (Fall, Sports, Transport and Other)

Results:

| Parameter | Neurlogical Level | DF | Estimate | Standard Error | Wald Chi-Square | Pr>ChiSq |
| --- | --- | --- | --- | --- | --- | --- |
| Intercept | Low | 1 | 1.4482 | 0.296 | 23.9424 | <.0001 |
| Intercept | Medium | 1 | 0.6083 | 0.2908 | 4.3749 | 0.0365 |
| Age | Low | 1 | -0.0436 | 0.00705 | 38.301 | <.0001 |
| Age | Medium | 1 | -0.0148 | 0.00633 | 5.4671 | 0.0194 |
| MOI_Fall | Low | 1 | 0.7595 | 0.2658 | 8.1622 | 0.0043 |
| MOI_Fall | Medium | 1 | 0.0605 | 0.2514 | 0.0579 | 0.8099 |

| R-Square | 0.0842 | Max-rescaled R-Square | 0.0948 |
| --- | --- | --- | --- |

**Table S5) Regression Analysis Results for Energy**

Methodology:

Multinomial Logistic Regression

Number of observations used:

532

Dependent variable:

- Energy (High, Low and Missing)

Independent variables:

- Age
- MOI (Fall, Sports, Transport and Other)

Results:

| Parameter | Energy | DF | Estimate | Standard Error | Wald Chi-Square | Pr>ChiSq |
| --- | --- | --- | --- | --- | --- | --- |
| Intercept | Low | 1 | -1.1263 | 0.3837 | 8.6163 | 0.0033 |
| Intercept | Missing | 1 | -1.4202 | 0.3972 | 12.7851 | 0.0003 |
| Age | Low | 1 | 0.038 | 0.00826 | 21.1781 | <.0001 |
| Age | Missing | 1 | 0.0485 | 0.00845 | 32.9444 | <.0001 |
| MOI_Other | Low | 1 | 2.052 | 0.5639 | 13.2422 | 0.0003 |
| MOI_Other | Missing | 1 | 0.1114 | 0.677 | 0.0271 | 0.8693 |
| MOI_Transport | Low | 1 | -4.0719 | 0.4925 | 68.3427 | <.0001 |
| MOI_Transport | Missing | 1 | -16.5542 | 201.7 | 0.0067 | 0.9346 |

| R-Square | 0.5436 | Max-rescaled R-Square | 0.6153 |
| --- | --- | --- | --- |

**Table S6) Regression Analysis Results for Injury Severity Score (ISS)**

Methodology:Ordinal Logistic Regression

Number of observations used:270

Dependent variable:

- ISS (High, Medium and Low)

Independent variables:

- Energy (High, Low and Missing)

Results:

| Parameter |  | Estimate | DF | Standard Error | Wald Chi-Square | Pr>ChiSq |
| --- | --- | --- | --- | --- | --- | --- |
| Intercept | Low | 0.1265 | 1 | 0.1823 | 0.4819 | 0.4876 |
| Intercept | Medium | 1.8784 | 1 | 0.2197 | 73.0734 | <.0001 |
| Energy_High |  | -1.522 | 1 | 0.2496 | 38.4115 | <.0001 |

| R-Square | 0.1416 | Max-rescaled R-Square | 0.1594 |
| --- | --- | --- | --- |

**Table S7) Regression Analysis Results for Glasgow Coma Scale (GCS)**

Methodology:Binomial Logistic Regression

Number of observations used:283

Dependent variable:GCS (GCS_13orless, GCS_14ormore)

Independent variables:

- Age
- Neurological Level (High, Medium and Low)
- Energy (High, Low and Missing)

Results:

| Parameter | DF | Estimate | Standard Error | Wald Chi-Square | Pr>ChiSq |
| --- | --- | --- | --- | --- | --- |
| Intercept | 1 | 1.8058 | 0.5959 | 9.1827 | 0.0024 |
| Age | 1 | 0.0320 | 0.0119 | 7.2269 | 0.0072 |
| NeuroLevel_High | 1 | -1.4442 | 0.3957 | 13.3233 | 0.0003 |
| Energy_High | 1 | -1.1677 | 0.4217 | 7.6688 | 0.0056 |

| R-Square | 0.0894 | Max-rescaled R-Square | 0.1573 |
| --- | --- | --- | --- |

**Table S8) Regression Analysis Results for ASIA Impairment Scale (AIS)**

Methodology:

Ordinal Logistic Regression

Number of observations used:489

Dependent variable:

- AIS (A, B, C and D)

Independent variables:

- Age
- Energy (High, Low and Missing)

Results:

| Parameter |  | DF | Estimate | Standard Error | Wald Chi-Square | Pr>ChiSq |
| --- | --- | --- | --- | --- | --- | --- |
| Intercept | D | 1 | -0.756 | 0.3044 | 6.1698 | 0.013 |
| Intercept | C | 1 | 0.0992 | 0.3028 | 0.1073 | 0.7432 |
| Intercept | B | 1 | 0.6354 | 0.304 | 4.3686 | 0.0366 |
| Age |  | 1 | 0.0119 | 0.00462 | 6.5746 | 0.0103 |
| Energy_Low |  | 1 | -0.7745 | 0.2252 | 11.8292 | 0.0006 |
| Energy_High |  | 1 | -1.1761 | 0.2214 | 28.2286 | <.0001 |

| R-Square | 0.0982 | Max-rescaled R-Square | 0.1061 |
| --- | --- | --- | --- |

**Table S9) Regression Analysis Results for Go To SCU**

Methodology:

Binomial Logistic Regression

Number of observations used:273

Dependent variable:GoToSCU (Yes, No)

Independent variables:

- Age
- MOI (Fall, Sports, Transport and Other)
- GCS (GCS_13orless, GCS_14ormore)
- AIS (A, B, C and D)

Results:

| Parameter | Estimate | DF | Standard Error | Wald Chi-Square | Pr>ChiSq |
| --- | --- | --- | --- | --- | --- |
| Intercept | -0.3063 | 1 | 0.4462 | 0.4712 | 0.4924 |
| Age | 0.0387 | 1 | 0.0097 | 15.9162 | <.0001 |
| MOI_Transport | 0.8952 | 1 | 0.355 | 6.3585 | 0.0117 |
| GCS_13orless | 2.0602 | 1 | 0.7668 | 7.2185 | 0.0072 |
| AIS_C | -1.4176 | 1 | 0.3805 | 13.8781 | 0.0002 |
| AIS_D | -1.3867 | 1 | 0.4045 | 11.7527 | 0.0006 |

| R-Square | 0.1658 | Max-rescaled R-Square | 0.2468 |
| --- | --- | --- | --- |

**Table S10) Regression Analysis Results for Mortality**

Methodology:Binomial Logistic Regression

Number of observations used: 482

Dependent variable: GoToMorgue (Yes, No)

Independent variables:

- Age
- GoToSCU (Yes, No)

Results:

| Parameter | Estimate | DF | Standard Error | Wald Chi-Square | Pr>ChiSq |
| --- | --- | --- | --- | --- | --- |
| Intercept | -8.8734 | 1 | 1.4634 | 36.7668 | <.0001 |
| Age | 0.0675 | 1 | 0.0148 | 20.6442 | <.0001 |
| SCU_Yes | 2.2669 | 1 | 1.0452 | 4.704 | 0.0301 |

| R-Square | 0.0729 | Max-rescaled R-Square | 0.2422 |
| --- | --- | --- | --- |

**Table S11) Regression Analysis Results for Length of Stay (LOS) in special care unit (SCU)**

Methodology: Linear Regression

Number of observations used: 204

Dependent variable:Log(LOS SCU)

Independent variables:

- Age
- Neurological Level (High, Medium and Low)
- GCS (GCS_13orless, GCS_14ormore)
- ISS (High, Medium and Low)
- AIS (A, B, C, D)

Results:

| Parameter | Estimate | DF | Standard Error | t Value | Pr>ChiSq |
| --- | --- | --- | --- | --- | --- |
| Intercept | 1.422 | 1 | 0.22474 | 6.33 | <.0001 |
| Age | 0.01204 | 1 | 0.00341 | 3.53 | 0.0005 |
| NeuroLevel_High | 0.42026 | 1 | 0.15537 | 2.7 | 0.0074 |
| NeuroLevel_Medium | 0.49543 | 1 | 0.15024 | 3.3 | 0.0012 |
| ISS_High | 0.43682 | 1 | 0.16367 | 2.67 | 0.0082 |
| ISS_Medium | 0.193 | 1 | 0.14675 | 1.32 | 0.19 |
| GCS_13orless | 0.7282 | 1 | 0.16366 | 4.45 | <.0001 |
| AIS_D | -0.6784 | 1 | 0.17211 | -3.94 | 0.0001 |

| R-Square | 0.2776 | Max-rescaled R-Square | 0.2518 |
| --- | --- | --- | --- |

**Table S12) Regression Analysis Results for LOS in Acute Care**

Methodology: Linear Regression

Number of observations used: 243

Dependent variable:Log(LOS Acute)

Independent variables:

- Age
- Neurological Level (High, Medium and Low)
- AIS (A, B, C, D)
- MOI (Fall, Sports, Transport and Other)
- GoToMorgue (Yes, No)
- ISS (High, Medium and Low)

Results:

| Parameter | Estimate |  | Standard Error | T Value | Pr>|t| |
| --- | --- | --- | --- | --- | --- |
| Intercept | -3.13841 | B | 3.93212 | -0.8 | 0.4256 |
| Age | 0.17126 |  | 0.02048 | 8.36 | <.0001 |
| NeuroLevel_High | -15.0629 | B | 0.80279 | -18.76 | <.0001 |
| NeuroLevel_Low | 10.22816 | B | 0.75636 | 13.52 | <.0001 |
| AIS_A | 16.33176 | B | 1.04606 | 15.61 | <.0001 |
| AIS_B | 17.11109 | B | 1.17423 | 14.57 | <.0001 |
| AIS_C | 15.84195 | B | 1.13232 | 13.99 | <.0001 |
| MOI_Sports | -6.73906 |  | 0.83311 | -8.09 | <.0001 |
| MOI_Fall | 1.80934 |  | 0.79380 | 2.28 | 0.0236 |
| DSCIC_Morgue_No | 9.85173 | B | 3.51448 | 2.8 | 0.0055 |
| ISS_High | 6.52156 | B | 0.76772 | 8.49 | <.0001 |
| ISS_Low | -4.41510 | B | 0.86435 | -5.11 | <.0001 |

| R-Square | 0.8845 |
| --- | --- |

**Table S13) Regression Analysis Results for Go to Rehab**

Methodology: Binomial Logistic Regression

Number of observations used:482

Dependent variable:GoToRehab (Yes, No)

Independent variables:

- Age
- AIS (A, B, C, D)
- Log(LOS Acute)

Results:

| Parameter | Estimate | DF | Standard Error | Wald Chi-Square | Pr>ChiSq |
| --- | --- | --- | --- | --- | --- |
| Intercept | 0.7374 | 1 | 0.3259 | 5.1216 | 0.0236 |
| Age | -0.0127 | 1 | 0.00562 | 5.1069 | 0.0238 |
| AIS_D | -1.2688 | 1 | 0.2371 | 28.6371 | <.0001 |
| log_LOS_Acute | 0.2325 | 1 | 0.0331 | 49.2393 | <.0001 |

| R-Square | 0.2342 | Max-rescaled R-Square | 0.3218 |
| --- | --- | --- | --- |

**Table S14) Regression Analysis Results for LOS in Rehabilitation Care**

Methodology: Linear Regression

Number of observations used:327

Dependent variable:

- Log(LOS Rehab)

Independent variables:

- AIS (A, B, C, D)
- Neurological Level (High, Medium, Low)
- Acute care admission (Yes, No)
- Log(LOS Acute)

Results:

| Parameter | Estimate | Standard Error | t Value | Pr>|t| |
| --- | --- | --- | --- | --- |
| Intercept | 3.95069 | 0.11654 | 33.9 | <.0001 |
| AIS_A | 0.889 | 0.09437 | 9.421 | <.0001 |
| AIS_B | 0.94132 | 0.11439 | 8.229 | <.0001 |
| AIS_C | 0.56567 | 0.09956 | 5.681 | <.0001 |
| NeuroLevel_Low | -0.33289 | 0.06807 | -4.89 | <.0001 |
| log_LOS_Acute | 0.33691 | 0.05245 | 6.423 | <.0001 |
| Acute_Yes | -1.29422 | 0.21748 | -5.951 | <.0001 |

| R-Square | 0.4509 | Max-rescaled R-Square | 0.4406 |
| --- | --- | --- | --- |

**Table S15) Regression Analysis Results for Discharge FIM**

Methodology: Linear Regression

Number of observations used: 164

Dependent variable:

- FIM

Independent variables:

- Age
- Discharge AIS (A, B, C, D, E)
- Neurological Level (High, Medium, Low)
- LOS Acute
- LOS Rehab
- AIS improved (Yes, No)

Results:

| Parameter | Estimate | Standard Error | Type II SS | F Value | Pr>F |
| --- | --- | --- | --- | --- | --- |
| Intercept | 129.1823 | 3.3263 | 280344 | 1508.28 | <.0001 |
| Age | -0.29661 | 0.06296 | 4125.23262 | 22.19 | <.0001 |
| NeuroLevel_Medium | -12.7481 | 2.71982 | 4083.38357 | 21.97 | <.0001 |
| NeuroLevel_High | -20.38725 | 2.96322 | 8798.32514 | 47.34 | <.0001 |
| DischargeAIS_D | 19.36536 | 2.82013 | 8764.37647 | 47.15 | <.0001 |
| DischargeAIS_E | 45.08859 | 14.32098 | 1842.45444 | 9.91 | 0.002 |
| LOS_Acute | -0.0053 | 0.00145 | 2503.11303 | 13.47 | 0.0003 |
| LOS _Rehab | -0.0887 | 0.01896 | 4067.21309 | 21.88 | <.0001 |
| AISImproved_Yes | -5.04314 | 2.3592 | 849.33539 | 4.57 | 0.0341 |

| R-Square | 0.7012 | Max-rescaled R-Square | 0.6858 |
| --- | --- | --- | --- |
